# Kinetics of angiogenic changes in a new mouse model for hepatocellular carcinoma

**DOI:** 10.1186/1476-4598-9-219

**Published:** 2010-08-20

**Authors:** Femke Heindryckx, Koen Mertens, Nicolas Charette, Bert Vandeghinste, Christophe Casteleyn, Christophe Van Steenkiste, Dominique Slaets, Louis Libbrecht, Steven Staelens, Peter Starkel, Anja Geerts, Isabelle Colle, Hans Van Vlierberghe

**Affiliations:** 1 Department of Gastroenterology and Hepatology, Ghent University Hospital, Ghent, Belgium; 2 Department of Nuclear Medicine, Ghent University Hospital, Ghent, Belgium; 3 Department of Gastroenterology, St. Luc University Hospital, Brussels, Belgium; 4 Medical Signal and Image Processing, Ghent University-IBBT, Ghent, Belgium; 5 Department of Morphology, Faculty of Veterinary science, Ghent University, Ghent, Belgium; 6 Laboratory of Radiopharmacy, Faculty of Pharmaceutical Sciences, Ghent University, Ghent, Belgium; 7 Department of Pathology, Ghent University Hospital, Ghent, Belgium

## Abstract

**Background:**

The increasing incidence of hepatocellular carcinoma in Western countries has led to an expanding interest of scientific research in this field. Therefore, a vast need of experimental models that mimic the natural pathogenesis of hepatocellular carcinoma (HCC) in a short time period is present. The goal of our study was (1) to develop an efficient mouse model for HCC research, in which tumours develop in a natural background of fibrosis and (2) to assess the time-dependent angiogenic changes in the pathogenesis of HCC.

**Methods:**

Weekly intraperitoneal injections with the hepatocarcinogenic compound N-nitrosodiethylamine was applied as induction method and samples were taken at several time points to assess the angiogenic changes during the progression of HCC.

**Results:**

The N-nitrosodiethylamine-induced mouse model provides well vascularised orthotopic tumours after 25 weeks. It is a representative model for human HCC and can serve as an excellent platform for the development of new therapeutic targets.

## Introduction

An efficient and representative mouse model is the cornerstone of a successful experiment. The growing incidence of hepatocellular carcinoma (HCC) in Western countries has resulted in an expanding interest of scientific research in this field. Therefore, a vast need of experimental models that mimic the natural pathogenesis of HCC in a short time period is present.

Several genetically modified mouse models (GMM) develop HCC in relatively short time periods. They often represent only one or a few specific mutation(s), while natural tumours are a dynamic environment consisting of a heterogenic cell population with different genotypes, which change over time as a response to variable external conditions [1-3]. Xenograft models are relevant for fast drug screening and proof-of-principle experiments [4], but face similar limitations as the GMMs, since only one cell phenotype is assessed, while tumours exist of a large variety of phenotypes. Results should always be interpreted with care, because introducing foreign cells in an animal system, as done in a xenograft mouse model, creates an altered physiological interaction between tumour and environment [5], leading to spectacular results that can seldom be confirmed in cancer patients [6].

A compound often used for the chemical induction of HCC is N-nitrosodiethylamine (DEN). DEN is metabolised by cytochrome P450 enzymes, which are abundantly present in the liver, leading to the formation of the reactive ethyl diazonium ion [7]. The latter holds the potential to alkylate DNA structures, causing alterations in the expression levels of tumour promoting and/or suppressing genes [8]. Single injections of DEN, sometimes in combination with phenobarbital treatment, are frequently used for the induction of HCC in mice and rats and have been validated as a genetically representative model for human HCC [9]. However, it does not induce fibrosis.

The goal of our study was (1) to develop an efficient mouse model for hepatocellular carcinoma (HCC) research, in which HCC develops in a natural background of fibrosis and (2) to assess the time-dependent angiogenic changes in the pathogenesis of HCC [10,11] since anti-angiogenic molecules are currently a hot topic in research concerning therapies for non-resectable HCC [12-14].

## Materials and methods

### Animals

4-week-old male mice (129S2/SvPasCrl) were purchased from Charles River laboratories (Brussels, Belgium). They were kept under constant temperature and humidity in a 12 h controlled dark/light cycle. Mice were fed *ad libitum *on a standard pellet diet. The Ethical Committee of experimental animals at the Faculty of Medicine and Health Sciences, Ghent University, Belgium, approved the protocols.

### HCC induction

5-week-old male mice (n = 45) received intraperitoneal injections once per week with DEN (35 mg/kg bodyweight) diluted in saline using a 0,5 mL syringe with a 29G needle. If mice suffered from weight loss ≥15% compared to the previous week, an injection was omitted. The control group was injected with an equal volume of saline and injections were randomly passed over in a comparable quantity as in the DEN-group.

### Tissue sampling & histology

After 4, 16, 20, 25 and 30 weeks, 8 animals per group were sacrificed under isoflurane (Forene^®^) anaesthesia while blood was obtained from the carotic artery. After macroscopic evaluation, all organs were sampled in 4% phosphate buffered formaldehyde (Klinipath, ref: 4078.9020) and embedded in paraffin. HCC-lesions and non-HCC-tissue were separately collected and snap frozen in liquid nitrogen. Haematoxilin-eosin staining (H&E) was performed to evaluate the morphological changes inflicted by the DEN-treatment. Sirius Red staining was carried out to score the fibrotic stage of the liver. Reticulin staining was performed to help identifying HCC-nodules. All stainings were done using standard histology protocols and evaluated by an experienced pathologist.

### Immunohistochemistry

Immunohistochemical stainings (IHC) were used to quantify protein expression levels inside HCC-nodules, tissue surrounding HCC-nodules and non-HCC tissue. As a marker for angiogenesis specific monoclonal antibodies were used against vascular endothelial growth factor (VEGF) (Santa Cruz biotechnology, ref sc-152), CD105 (R&D systems, ref AF1320) and Tie2 (BD Bioscience, ref 557039). Tumour hypoxia was evaluated by staining for hypoxia inducible factor 1 alpha (HIF1α) (Santa Cruz ref sc-53546) and macrophages were visualised using F4/80-staining (AbD serotec, ref MCA497G). A negative marker for HCC, Fatty Acid Binding Protein (FABP, Hycult biotechnology, ref HP8010) was used [15]. Stainings were performed as previously described [15-17] and were semi-quantitatively measured by Olympus Cell^D ^software. Intercapillary distance (ICD) was used as a marker for microvessel density, by measuring the average distance between vessels in HCC-nodules on CD105-stained slides.

### Medical imaging

Additional *in vivo *tests were performed using microPET imaging as non-invasive technology. The latter acquisitions were performed using a GE FLEX Triumph micro-PET/SPECT/CT scanner (Gamma Medica-Ideas). This state-of-the-art scanner consists of a micro-PET module (LabPET8) with 2'2'10 mm^3 ^LYSO/LGSO scintillators in an 8-pixel, quad-APD detector module arrangement. This system is capable of delivering 1 mm spatial resolution in rodents at a sensitivity of 4%, thereby covering a field-of-view of 10 cm transaxially by 8 cm axially, while the CT part can scan structures down to 10-15 μm. The micro-CT part consists of a high-resolution micro-CT tube with a focal spot size switchable between 10 or 50 μm, combined with a flat-panel CsI detector.

Animals were injected with 18,5 mBq of [18F]- fluoromethylcholine ([18F]FMCH) (Laboratory of Radiopharmacy, Ghent, Belgium) [18,19] immediately prior to their microPET scan on the camera bed at the start of a dynamic acquisition. Frames of 15×20 sec and 5×5 min were accordingly sequentially recorded. For anatomical localisation, a microCT-scan was sequentially acquired using 256 projections over 360 degrees at 70 kVp/180 μA and 1,3 magnification with a spot size of 50 μm. This results in a scanning time below 2 minutes, whilst keeping the radiation dose at 20 - 25 mGy. This radiation dose is sufficiently low enough to allow for follow-up studies. The resulting PET data were reconstructed using 30 iterations of the Maximum Likelihood Expectation Maximisation algorithm in 160×160×63 images of 0,5 × 0,5 × 1,175 mm voxel size and no *posteriori *3D filtering was applied. CT-reconstruction was straightforward analytical. All images were fused and analysed with VIVID (Amira ^®^, San diego, USA).

Vascular corrosion casts (VCC) were obtained by perfusing Batson n°17 (Polysciences ref: 07349) through the aorta (arterial casts) or vena ileocolica (venous cast); and dissolving soft tissue in KOH. The VCC were then scanned in the micro-CT scanner, after which the reconstructed dataset was segmented before generating 3D surface models. In order to assess the VCC with scanning electron microscopy (SEM), the relevant parts were cut off and coated with platinum.

### Protein expression

Alterations in protein expression levels of angiogenic factors in HCC-tissue were measured by ELISA [20]. Placental growth factor (PlGF) (Mouse PlGF-2 Quantikine ELISA Kit, R&D Biosystems) and VEGF (Mouse VEGF Quantikine ELISA Kit, R&D Systems) were measured in liver tissue and in serum. Protein levels of soluble VEGF receptor 1 (sVEGFR1) (Mouse sVEGF R1/Flt-1 Quantikine ELISA Kit R&D Biosystems) were measured in serum. To normalise the total protein concentration in the samples, a normalisation factor was determined by a Biorad RC/DC Protein Assay kit.

### Statistics

Data were statistically analysed with SPSS 16. Datasets were tested for normality using the Kolmogorov-Smirnov test before further analysis. Parametric data were subjected to a student's t-test to evaluate the difference between the DEN and control group. Data that did not show a normal distribution were tested with the non-parametric Mann-Whitney-U test. A *p *value of < 0.05 was considered statistically significant. Correlations were calculated using the Pearson correlation test.

## Results

### Macroscopic evaluation

**Figure 1:**
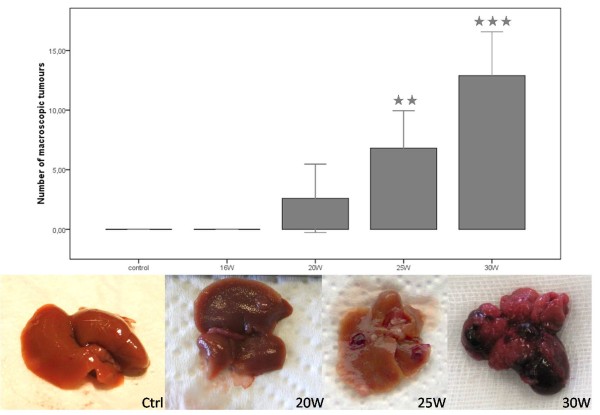
**Macroscopic HCC-lesions at different time points throughout the experiment**. Asterisks (*) represent the significant P-values of the control group compared to DEN-groups (** = p < 0,01 and *** = p < 0,001).

Significant lower weight (p < 0,001) was observed between adult DEN-mice (25,26 g (± 0,49)) and control mice (31,28 g (± 0,91)) at 25W. Macroscopic evaluation of the liver revealed tumours at 20W (2,6 (± 2,66) tumours/liver), 25W (6,81 (± 1,69) tumours/liver) and 30W (12,9 (± 2,17) tumours/liver) (figure 1).

### Microscopic evaluation

#### HCC progression

**Figure 2:**
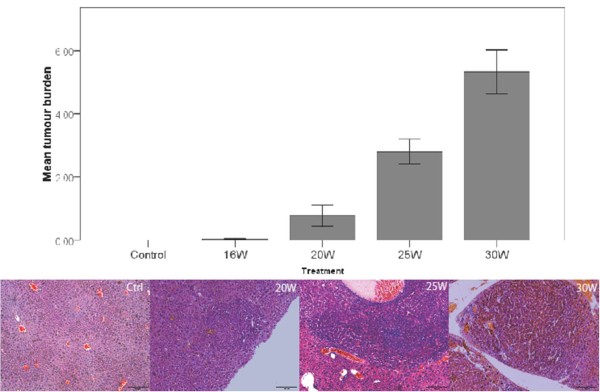
**Mean HCC burden (size × number tumours) at different time points and representative pictures of H&E stained HCC-nodules**.

**Figure 3:**
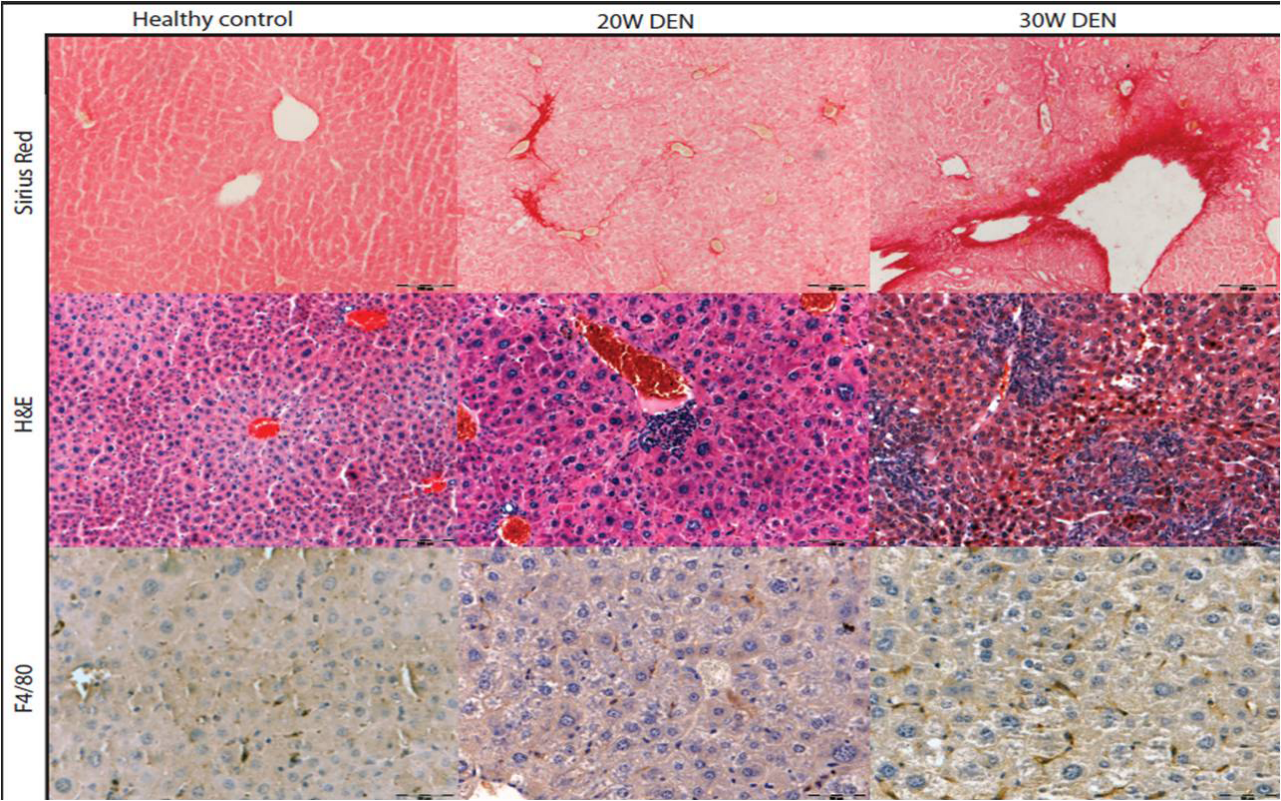
**Representative pictures of fibrosis in non-tumorous tissue on sirius red staining (top), inflammatory foci on H&E staining (middle) and macrophages on F4/80 immunohistochemistry (bottom)**.

H&E staining showed dysplastic changes at 20W, 25W and 30W. Small cell dysplasia was frequently found throughout the liver and readily distinguishable nodules of neoplasia were seen at 25W and 30W (figure 2). These nodules were confirmed as HCC using reticulin staining and HE (and immunohistochemistry for FABP). HCC nodules were identified and measured on reticulin and H&E stained histological slides. Tumour burden (size × number) increased significantly during DEN-treatment (figure 2). Sirius red staining demonstrated the fibrotic action of DEN, with time-dependent increase of Metavir-score (figure 3), while controls did not show any fibrotic septa.

**Figure 4:**
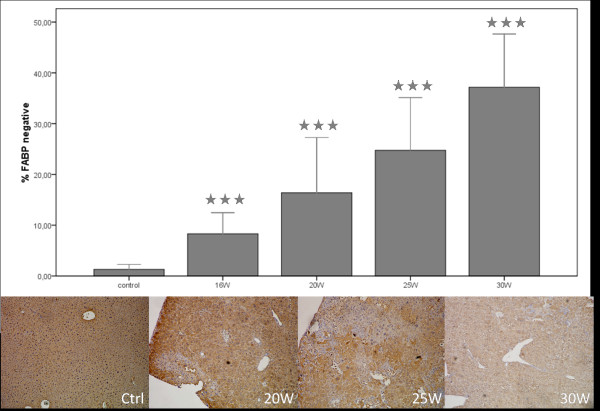
**Percentage of FABP-negative sites and representative pictures of the IHC staining**. Asterisks (*) represent the significant p-value of the control group compared to DEN-groups (*** = p < 0,001).

A time-dependent increase in FABP-negative spots was seen throughout the experiment (figure 4). While parts of the liver were FABP-negative, some hepatocytes had an increased FABP-expression in the DEN-treated groups whereas control livers showed a homogenous expression (figure 4). FABP (p < 0,001) was significantly down regulated in all the DEN-treated groups compared to the control group (figure 4).

#### Inflammation and fibrosis

**Figure 5:**
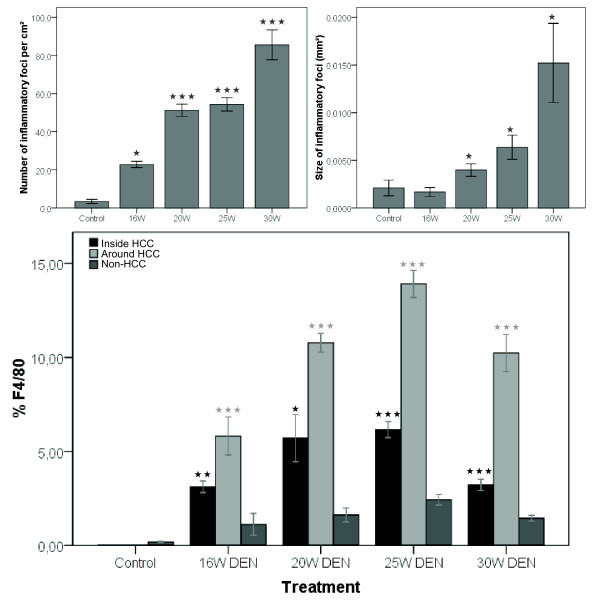
**Top: mean number (left) and size (right) of inflammatory foci at different time points; Bottom: expression of F4/80 on immunohistochemistry.** Asterisks (*) represent the significant P-value of the difference between expression inside and around HCC-nodules compared to non-tumour tissue in DEN-treated livers (* = p < 0,05, ** = p < 0,01, *** = p < 0,001).

The number and size of inflammatory foci significantly increases after 20W (p < 0,001 and p < 0,05), 25W (p < 0,001 and p < 0,05) and 30W DEN (p < 0,001 and p < 0,05) compared to control livers (figure 3 and 5). This was accompanied by an increased abundance of Kuppfer cells in and around HCC, as well as in non-HCC tissue (figure 3). Compared to non-HCC tissue, the expression of F4/80 was significantly up-regulated after 16W (p < 0,01), 20W (p < 0,05), 25W (p < 0,001) and 30W (p < 0,001) in HCC, and in the surrounding matrix of HCC-lesions (p < 0,001) (figure 5).

#### Angiogenesis

**Figure 6:**
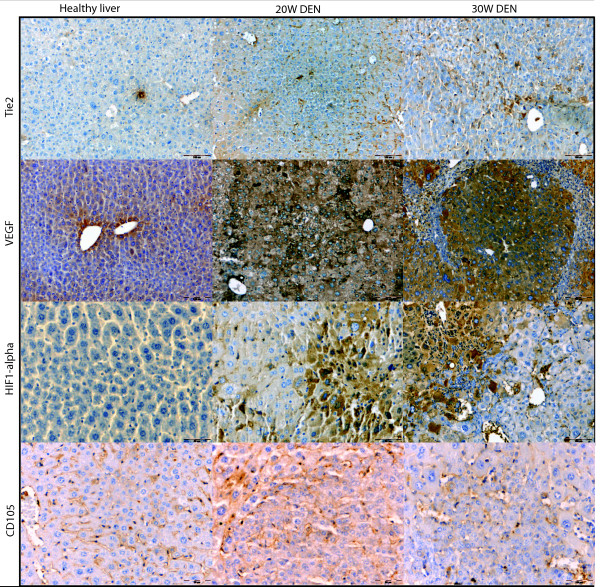
**Representative pictures of immunohistochemical stainings for Tie2, VEGF, HIF1α and CD105 in healthy liver, 20W DEN (tumour tissue) and 30W DEN (tumour tissue)**.

**Figure 7:**
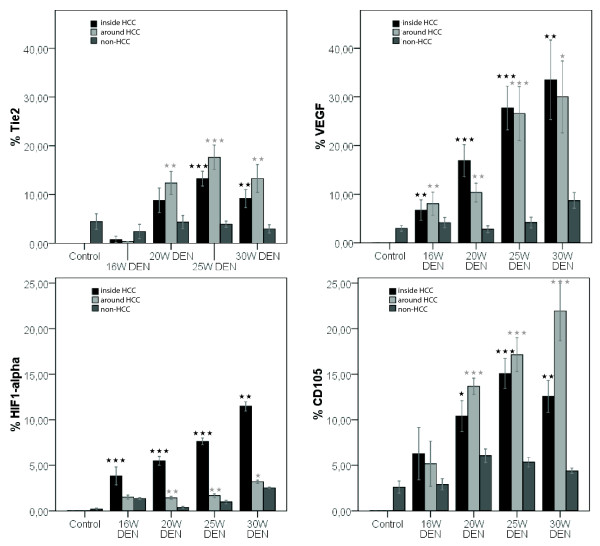
**Top: Tie2 epxression (left) and VEGF expression (right); Bottom: HIF1-α (left) and CD105 (right).** Asterisks (*) represent the significant P-value of the difference between expression inside and around HCC-nodules compared to non-tumour tissue in DEN-treated livers (* = p < 0,05, ** = p < 0,01, *** = p < 0,001).

**Figure 8:**
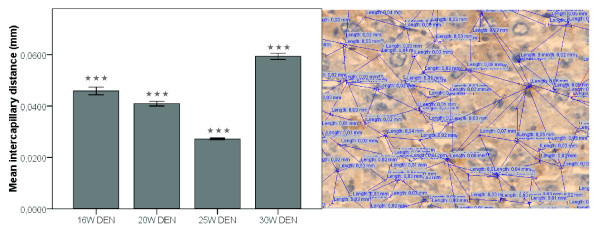
Left: mean intercapillary distances at different time points,*** = p < 0,001, right: measurement of ICD.

VEGF expression was more promintent inside HCC-nodules (figure 6 and 7) than in the surrounding matrix, yet both were significantly increased (p < 0,01) after 20W, 25W and 30W DEN compared to adjacant non-HCC tissue. VEGF-levels were significantly increased inside HCC-nodules compared to controls after 25W (p < 0,05) and 30W DEN (p < 0,05) (table 1). The expression of VEGF was correlated (r = 0,68, p < 0,001) with HIF1-α levels. HIF1α levels were significantly up-regulated in and around tumour tissue at 20W (p < 0,001), 25W (in: p < 0,001; around: p < 0,05) and 30W DEN (p < 0,001) compared to adjacant non-HCC tissue (figure 6 and 7). Non-HCC tissue was hypoxic after 16W (p < 0,01), 25W (p < 0,01) and 30W (p < 0,001) of DEN compared to healthy controls (table 1). The increased HIF1α expression was more prominent inside HCC nodules than in surrounding matrix (figure 6), the latter being characterised by a major increase in vascularisation after 20W, 25W and 30W of DEN compared to non-tumour tissue (p < 0,001). CD105 was also increased inside HCC-lesions after 20W (p < 0,05), 25W (p < 0,001) and 30W (p < 0,01) (figure 6 and 7), implicating an increased intratumoral neovascularisation, confirmed by ICD (figure 8). The ICD decreased at 20W and 25W, verifing neovascularisation; an increase was seen at 30W (figure 8). Differences of ICD were statistically significant between all groups (p < 0,001). DEN-induced HCC-lesions showed an increased expression of Tie2 which was higher in HCC-surrounding tissue than inside HCC-nodules (figure 6). Tie2 was significantly up-regulated around HCC after 20W (p < 0,01), 25W (p < 0,001) and 30W (p < 0,01) compared to non-HCC tissue, and inside HCC after 25W (p < 0,001) and 30W DEN (p < 0,01 (figure 7, table 1). Tie2 expression was correlated (r = 0,58, p < 0,001) with macrophage recruitment (F4/80-staining).

**Table 1:** Average protein expression in DEN-treated liver determined with immunohistochemistry by measuring DAB-positive staining

	Inside tumour											
	16W			20W			25W			30W		
	Av (%)	SEM	*p*	Av (%)	SEM	*p*	Av (%)	SEM	p	Av (%)	SEM	*p*
TIE2	0,75	0,68	*NS*	8,81	2,52	*NS*	13,24	1,53	*< 0,01*	9,17	1,84	*NS*
VEGF	6,74	2,09	*< 0,05*	16,91	3,28	*< 0,001*	27,72	3,42	*< 0,001*	33,50	8,16	*< 0,01*
HIF1-a	3,79	0,57	*< 0,001*	5,49	0,48	*< 0,001*	7,63	0,37	*< 0,001*	11,47	0,50	*< 0,001*
CD105	6,29	2,88	*NS*	10,40	1,70	*< 0,001*	15,08	1,44	*< 0,001*	12,57	1,75	*< 0,001*
F4/80	3,12	0,30	*< 0,001*	5,70	1,25	*< 0,001*	6,16	0,46	*< 0,001*	3,21	0,30	*< 0,001*

	**Surrounding tumour**											

	16W			20W			25W			30W		
	Av (%)	SEM	*p*	Av (%)	SEM	*p*	Av (%)	SEM	p	Av (%)	SEM	*p*
TIE2	0,36	0,68	*NS*	12,35	2,37	*< 0,05*	17,63	2,52	< 0,01	13,30	2,89	*< 0,05*
VEGF	8,06	2,09	*< 0,01*	10,35	1,92	*< 0,001*	26,56	4,23	< 0,001	30,01	7,39	*< 0,001*
HIF1-a	1,51	0,70	*< 0,01*	1,43	0,17	*< 0,001*	1,67	0,19	< 0,001	3,20	0,17	*< 0,001*
CD105	5,17	2,49	*NS*	13,68	0,88	*< 0,001*	17,14	1,77	< 0,001	21,94	3,24	*< 0,001*
F4/80	5,82	0,37	*< 0,001*	10,78	0,50	*< 0,001*	13,92	0,72	< 0,001	10,24	0,98	*< 0,001*

	**Non-tumour**											

	16W			20W			25W			30W		
	Av (%)	SEM	*p*	Av (%)	SEM	*p*	Av (%)	SEM	p	Av (%)	SEM	*p*
TIE2	4,77	1,75	*NS*	4,33	1,36	*NS*	3,90	0,62	NS	2,95	0,83	*NS*
VEGF	4,50	0,88	*NS*	2,80	0,73	*NS*	4,19	0,85	NS	8,72	1,60	*< 0,01*
HIF1-a	1,04	0,16	*< 0,01*	0,38	0,12	*NS*	0,98	0,19	< 0,01	2,53	0,08	*< 0,001*
CD105	3,74	0,68	*NS*	6,06	0,74	*< 0,05*	5,36	0,48	< 0,001	4,40	0,31	*< 0,05*
F4/80	0,87	0,27	*NS*	1,62	0,37	*< 0,05*	2,43	0,28	< 0,01	1,44	0,16	*< 0,01*

P-values were calculated for the difference with healthy liver protein levels.

### Medical imaging

**Figure 9:**
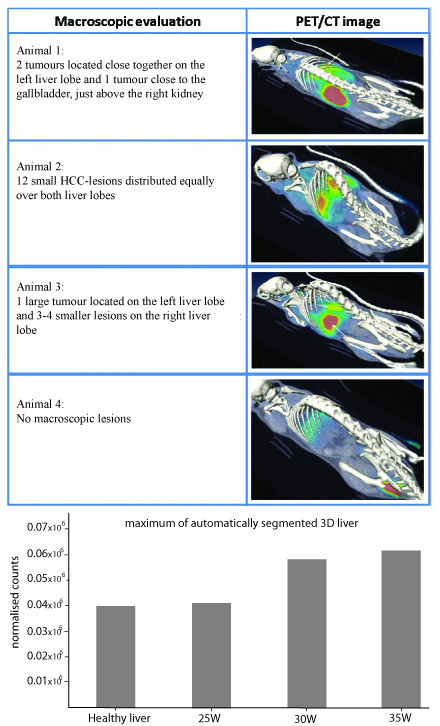
**Top: PET-images of mice at 30 weeks, static reconstruction of a 30 min acquisition;** Bottom: normalised counts of F[18]MCH uptake during a follow-up study.

Preliminary results, illustrated by Figure 9 suggest that these tumours could be visualised by [18F]FMCH PET imaging; and that this technique is useful for follow up studies. A short follow study (25W - 35W) was conducted on mice. An increased uptake was seen at the sequential measurements, which correlates with their increased tumour burden. However, further confirmation in larger groups is needed.

**Figure 10:**
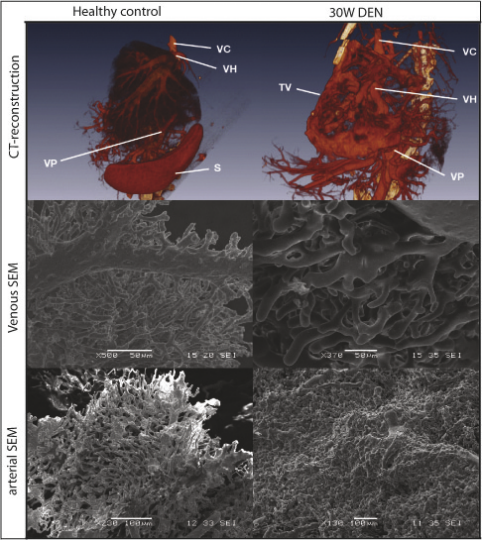
**Top: 3D reconstructions of vascular corrosion castings**, **Middle**: **SEM-images of venous vascular corrosion castings; **Bottom**: SEM-images of arterial vascular corrosion castings.**

**Figure 11:**
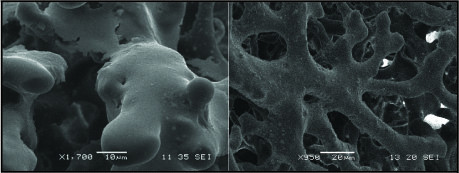
**Left: sprouting angiogenesis; Right: intussusceptive angiogenesis**.

The combination of VCC and CT-scanning revealed the chaotic pattern and dishierarchical organisation of tumour vessels (figure 10). The SEM-images provide further insight in the microvasculature (figure 10), showing the tumour vessel abnormality and visualising two mechanisms of neovascularisation, pruning and intussusceptive angiogenesis (figure 11).

### Protein expression

**Figure 12:**
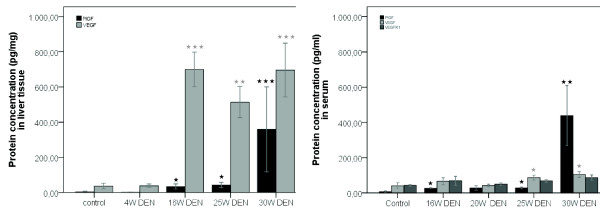
**Left: protein concentrations in liver tissue, determined by ELISA**; **Right: protein concentrations in serum, determined by ELISA**. Asterisks (*) represent the significant P-value of the difference between DEN-treated groups and controls. (* = p < 0,05, ** = p < 0,01, *** = p < 0,001).

PlGF and VEGF are important factors involved in tumour angiogenesis and were significantly up-regulated in liver tissue at 16W (respectively p < 0,05 and p < 0,001), 20W (p < 0,05 and p < 0,01) and tumour tissue samples at 25W DEN (p < 0,05 and p < 0,01). The up-regulation of PlGF was more prominent in tumour tissue at 30W with an average of 422,28 pg/mg (± 76,99) PlGF compared to the 2,90 pg/mg (± 0,77) PlGF detected in the control group (p < 0,001) (figure 12).

In serum, the PlGF protein levels were significantly up-regulated at 16W (p < 0,05), 25W (p < 0,05) and 30W (p < 0,01) with similar results as seen in liver tissue (figure 12). VEGF levels were up-regulated at 25W (p < 0,05) and 30W (p < 0,05). There was a slight increase of sVEGFR1 at 30W DEN treatment, but no significant difference was seen between controls and 25W DEN (figure 12).

## Discussion

Animal models can provide essential knowledge about the pathogenesis of hepatocellular carcinoma, particularly when they mimic the tissue environment in which human tumours develop. N-nitrosodiethylamine has been shown to induce tumours which are molecularly similar to human HCC with poor prognosis [9]. Yet, the major drawback is the long time needed for tumour progression, it can take over one year for tumours to develop [21-26]. The macroscopic and microscopic evaluation of tumours, PET-CT images and histopathological analysis confirmed the presence of HCC after 25 weeks. While most DEN-induced models take at least 1 year to develop tumours, weekly intraperitoneal injections with 35 mg/kg DEN give rise to relatively fast tumour occurrence in a background of fibrosis. Since HCC is known to be a hypervascularised tumour, and recent therapies have focused on inhibiting angiogenesis, we have studied the time-dependent changes in angiogenic and inflammatory factors in this mouse model.

Previous studies with several DEN-induced rat and mice models, have shown that the expression level of FABP is decreased in DEN-induced liver lesions [15,27]. These L-FABP-negative foci are correlated with a higher growth potential. Consequently, the FABP-expression was measured as a parameter for HCC-development. Even after a relatively short period of DEN administration (16W) significant decrease in FABP-expression is seen compared to controls. After 25W DEN large FABP-negative lesions appear throughout the liver, alternating with small FAPB-positive regions, while control tissue showed a homogenous expression.

DEN-injections also gave rise to increased inflammation, confirmed by H&E staining, as well as F4/80 which is a marker for Kuppfer cells. Activation of Kupffer cells leads to the release of cytokines, reactive oxygen species, and platelet activating factors, which are involved in the angiogenic switch. Furthermore, the chronic inflammation causes hepatocyte damage, leading to fibrosis as seen on the sirius red staining. In our study we show that Tie2 is up-regulated in the fibrotic matrix surrounding the HCC-lesions, which was also characterised by increased macrophage infiltration, confirming Tie2's correlation with hepatic inflammation. In addition, Tie2 is a receptor for angiopoetins, which play an important role in angiogenesis. Furthermore, the presence of fibrosis creates an increased intrahepatic vascular resistance and impairs oxygen diffusion, resulting in hypoxia which is the onset for the angiogenic switch. This is supported by our findings HIF1α and VEGF levels were increased in the fibrotic non-HCC tissue after DEN-treatment. The increased expression of HIF1α inside tumour nodules, confirms the vast oxygen need of the malignant hepatocytes. To fulfil that requirement, tumour cells start expressing angiogenic factors, such as VEGF and PlGF, inducing the angiogenic switch.

Up till now, VEGF was one of the key targets in angiogenesis research, but recently PlGF gained interest because its association with pathological conditions. PlGF levels are known to be elevated in a variety of cancers [28-33] and is associated with poor prognosis in HCC [34]. The up-regulation of PlGF in this DEN-model supports the theory that PlGF plays an essential role in the angiogenesis of HCC. Protein levels of sVEGFR1, an important inhibitor for VEGF and PlGF, did not significantly alter, suggesting that the increase of angiogenic factors was physiologically relevant. Thus, the increase of angiogenic factors (VEGF, PlGF and Tie2), resulted in an increased neovascularisation. The IHC results of the CD105-staining revealed a low CD105 expression in the normal endothelial cells of the liver and was up-regulated in the malignant liver lesions at 20W, consistent with previous studies [17]. The intercapillary distances significantely decreased after 25W DEN, verifying neovascularisation; although an increase was seen at 30W supporting the theory that malignant hepatocytes tend to outgrow their vascular supply and become accostumed to hypoxia. This up-regulation of CD105 was more prominent around HCC-lesions, suggesting that the newly formed vessels form a circumferential mantle around the tumours. This was confirmed by the CT-reconstructions of the vascular casts. SEM-images demonstrate the presence of neo-angiogenesis, by visualising budding and intussusceptive angiogenesis. Furthermore, the images illustrate the overall microvascular abnormality of DEN-treated livers. Thus, the combination of casting and micro-CT imaging provides unique data on the hepatic circulation and neovascularisation. It allows to digitally visualise the complex architecture of the liver blood vessels and to provide high resolution data for qualitative morphological analysis.

During this study we have established a new mouse model for HCC, which is considerably faster than current chemically induced models and has the advantage of tumour progression occurring in a background of inflammation and fibrosis. Furthermore, angiogenic factors were assessed at different time points, to provide important information about the kinetic changes of angiogenic factors during HCC progression. Moreover, several innovative imaging techniques were applied, not only to assess tumour growth, but also to provide further insight in microvascular alterations HCC livers.

## Competing interests

The authors declare that they have no competing interests.

## Authors' contributions

FH has done the DEN-injections, sacrifications, histology, ELISAs, immunohistochemistry, statistical analysis and the writing of the manuscript. KM was responsible for planning the PET-scans and evaluation of images, he also contributed to the medical imaging results in the draft. CC was responsible for producing the vascular corrosion casts and B.V. made 3D CT-reconstructions. NC & PS did preliminary research on the DEN-model and FABP-staining, both contributed to the discussion. CVS was responsible for the practical training of FH and assisted during sacrifications, furthermore he provided ready-to-use protocols for immunohistochemistry. DS is responsible for the production of radiolabelled choline. LL evaluated the histopathology of the slides at different time points and contributed to the histology-part of the manuscript. SS is responsible for the small animal imaging facility, he has coordinated the scans and did the PET-CT-constructions, he added the technical information concerning the scans to the material and method section. IC and HVV helped with the study design and are "the scientific mentors" of the first author. All authors have read and approved the final manuscript.
